# Kobuvirus

**DOI:** 10.3201/eid1510.999999

**Published:** 2009-10

The Japanese term *kobu* (kō′ bü), meaning hump or knob, was chosen to designate this genus in the family *Picornaviridae* because of the distinctive icosahedral structure of the viruses in this group. The genus contains the species *Aichi virus* and *Bovine kobuvirus*, and a candidate porcine kobuvirus has been identified. *Aichi virus*, the type species, was first recognized in 1989 as the cause of oyster-associated nonbacterial gastroenteritis in persons in the Aichi Prefecture in Japan.

